# Ten years follow-up after surgery for a foveal detachment due to optic disc pit: a case report of outer retinal layer healing

**DOI:** 10.1186/s12886-017-0626-9

**Published:** 2017-12-04

**Authors:** Audrey Giocanti-Aurégan, Elige A. Chbat, Christophe G. H. Morel, Bruno R. Morin, John G. Conrath, François C. Devin

**Affiliations:** 1 Ophthalmology department, Avicenne hospital, 125 rue de Stalingrad, 93000 Bobigny, France; 2 Monticelli-Paradis Center, 13008 Marseille, France

**Keywords:** Foveal detachment, Optic disc pit, Photoreceptor regeneration, Retinal pigment epithelium, Case report

## Abstract

**Background:**

To report a case of complete progressive visual recovery and healing of outer retinal layers after vitrectomy for foveal detachment associated with optic disc pit.

**Case presentation:**

Optical coherence tomography (OCT) follow-up was performed on a 15-year-old boy with deep optic disc pit and foveal detachment, before and for 10 years after vitrectomy with gas. The foveal detachment was successfully reattached with complete reapplication of the retina. OCT scans showed a progressive long-term retinal healing with reappearance of the ellipsoid line and visual acuity improved from 20/100 before surgery to 20/25, 10 years after surgery.

**Conclusions:**

Photoreceptor regeneration after foveal detachment surgery has been already described only in zebrafish but never humans. However, we highlight with this case that in humans, a healing process of the outer retinal layers can occur with reappearance of the ellipsoid zone on OCT. This healing process may take several years and allow a complete functional restoration.

## Background

A few cases of outer retinal layer reapperance have been reported previously after macular hole [1] or macula-off retinal detachment surgery [2, 3]. However, the underlying mechanisms are still not well established. Photoreceptors regeneration have only been demonstrated in zebrafish [4], but never humans. To date, the main hypothesis of outer retinal layer reappearance after macula-off retinal detachment or macular hole is a healing process of the outer retina with a reorganization of the remaining photoreceptors.

## Case presentation

**Fig. 1:**
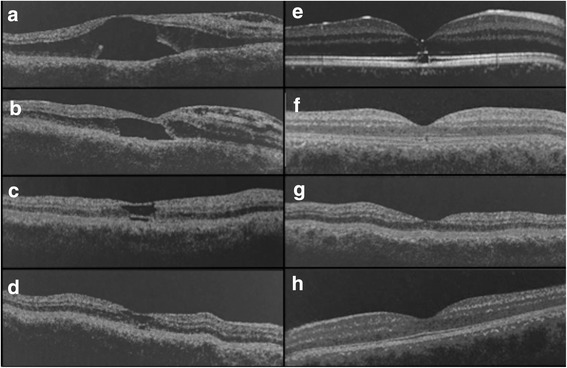
Horizontal optical coherence tomography B-scans (OCT) showing progressive recovery from outer retina disruption due to foveal detachment associated with optic disc pit. **a** Pre-operative Time-domain OCT showing foveal detachment in 2005. Visual acuity was 20/100. **b** 6 months after surgery, reapplication of the edges of the foveal detachment but no outer retina was visible at the center of the retina. The visual acuity was 20/40. **c**, **d** On Time-domain OCT, 1 (**c**) and 3 years (**d**) after surgery, the area of outer retina disorganization decreased and the ellipsoid zone has returned to normal except at the very center. Visual acuity improved to 20/32 at both timepoints. **e** Spectral-domain OCT showing a focal alteration of the ellipsoid zone 5 years after surgery, and (**f**) OCT scan showing the perfect reconstitution of the outer retina with enhanced outer layer density and a visual acuity of 20/25, 10 years after surgery. The central retinal thickness is 245 μm. **g** Left eye is unremarkable on Time-domain OCT at baseline and on Spectral-domain OCT 10 years post surgery (**h**). The central retinal thickness is 275 μm

A 15 year-old boy with no past medical history presented for a vision loss of the right eye. The patient took no medication. There was no significant medical family history. His best-corrected visual acuity was 20/100 for the right eye, and 20/20 for the left. The intraocular pressure was normal bilaterally. The pupils were equal, round, and reactive. A slit-lamp examination of the anterior segment was normal for both eyes without evidence of iris coloboma. A macular examination revealed an absence of the foveal reflex on the right eye while present on the left eye. Time-domain optical coherence tomography (OCT) revealed a foveal detachment related to an optic disc pit on the right eye. There was an incomplete macular hole associated with detachment of the boundaries of the macular hole, with loss of the outer retina (Fig. 1a). The patient underwent a pars plana vitrectomy with gas tamponade (C2F6) of his right eye. The post-operative OCT scans at 6 months showed a wide disorganization of the outer retinal layers with a partial reapplication of the retina (Fig. 1b). Visual acuity improved to 20/40 at 6 months post surgery. By 1 year, the visual acuity has improved to 20/32. On OCT scans, a slight disorganization of the photoreceptor layer remained underneath the right fovea at one and 3 years (Fig. 1c, d). At 5 years post-surgery, a focal interruption of the ellipsoid zone (EZ) remained (Fig. 1e). At 10 years follow-up after surgery, his visual acuity has improved to 20/25 and complete restoration of the EZ and the cone interdigitation zone (CIZ) at the fovea was observed (Fig. 1f). OCT of the left eye was unremarkable (Fig. 1g, h **).**

## Discussion and conclusions

Spectral-domain optical coherence tomography allows a direct visualization of in vivo retinal morphology and provides informations of the structural postoperative macular changes.

The reappearance of outer retinal layers has been already shown to be associated with a good visual acuity recovery following surgery for rhegmatogenous retinal detachment [2]. During this reappearance phase, the thickness of the EZ-Retinal pigmented epithelium complex and cone density increase, leading to the formation of foveal bulge and good vision after successful reattachment of macula-off rhegmatogenous retinal detachment [2]. The visual recovery is particularly described in the literature over the first year after retinal detachment surgery and associated with restoration or not of the EZ or CIZ.

Here the originality of our case is the long term follow-up over 10 years hightlighting the possibility after the first year following surgery, to still observe a gain in vision and a complete restoration of the macular anatomy on OCT scans in case of macular detachment due to optic pit reapplication. We described the outer retinal healing after surgery in this particular indication of foveal detachment due to optic disc pit for the first time, to the best of our knowledge. Our patient was 15-year old at the time of surgery, this young age could suggest probably a higher susceptibility for his retina to heal. The central retinal thickness slightly thicker in the left eye at the end of follow-up comforts also this healing process hypothesis with photoreceptors reorganization.

This case corroborates the possibility already known of photoreceptor reorganization and outer retinal layer healing after surgery in a yet non described indication of macular detachment due to an optic disc pit in a 15 year-old boy. Here we highlight that this healing process may take several years to occur.

## Abbreviations

(CIZ): Cone interdigitation zone
(EZ): Ellipsoid zone
(OCT): Spectral-domain optical coherence tomography

## References

[1] Lee JE, Lee SU, Jea SY (2008). Reorganization of photoreceptor layer on optical coherence tomography concurrent with visual improvement after macular hole surgery. Korean J Ophthalmol KJO.

[2] Kobayashi M, Iwase T, Yamamoto K (2016). Association between photoreceptor regeneration and visual acuity following surgery for rhegmatogenous retinal detachment. Invest Ophthalmol Vis Sci.

[3] Sridhar J, Flynn HW, Fisher Y (2015). Inner segment ellipsoid layer restoration after macula-off rhegmatogenous retinal detachment. Ophthalmic Surg Lasers Imaging Retina.

[4] Fraser B, DuVal MG, Wang H, Allison WT (2013). Regeneration of cone photoreceptors when cell ablation is primarily restricted to a particular cone subtype. PLoS One.

